# Cellular and molecular mechanisms involved in the establishment of HIV-1 latency

**DOI:** 10.1186/1742-4690-10-11

**Published:** 2013-02-01

**Authors:** Daniel A Donahue, Mark A Wainberg

**Affiliations:** 1 McGill University AIDS Centre, Lady Davis Institute, Jewish General Hospital, Montreal, Québec, Canada; 2 Department of Microbiology and Immunology, McGill University, Montreal, Québec, Canada

**Keywords:** Latency, CD4 T-cell, Reservoir, Establishment, Transcriptional interference, Epigenetics, Chromatin

## Abstract

Latently infected cells represent the major barrier to either a sterilizing or a functional HIV-1 cure. Multiple approaches to reactivation and depletion of the latent reservoir have been attempted clinically, but full depletion of this compartment remains a long-term goal. Compared to the mechanisms involved in the maintenance of HIV-1 latency and the pathways leading to viral reactivation, less is known about the establishment of latent infection. This review focuses on how HIV-1 latency is established at the cellular and molecular levels. We first discuss how latent infection can be established following infection of an activated CD4 T-cell that undergoes a transition to a resting memory state and also how direct infection of a resting CD4 T-cell can lead to latency. Various animal, primary cell, and cell line models also provide insights into this process and are discussed with respect to the routes of infection that result in latency. A number of molecular mechanisms that are active at both transcriptional and post-transcriptional levels have been associated with HIV-1 latency. Many, but not all of these, help to drive the establishment of latent infection, and we review the evidence in favor of or against each mechanism specifically with regard to the establishment of latency. We also discuss the role of immediate silent integration of viral DNA versus silencing of initially active infections. Finally, we discuss potential approaches aimed at limiting the establishment of latent infection.

## Review

Latently infected cells represent the major obstacle to either a sterilizing or a functional HIV-1 cure. HIV-1 latency can be defined as a reversibly nonproductive infection of a cell [1], which is usually interpreted to refer to an integrated provirus that is replication-competent but transcriptionally silent. In light of recent evidence, this definition might be expanded to include proviruses that express some but not all gene products in the absence of virion production [2-5]. The latent reservoir is established very early after infection [6,7], and reactivation of latently infected cells serves as a major source of viral rebound upon treatment failure [8,9]. Recent studies of the dynamics of viral load decay have shown the presence of two kinetically distinct latent reservoirs, *i.e.* the sources of plasma viremia during the third and fourth phases of decay [7,10,11], potentially representing different memory CD4 T-cell subsets. Multiple approaches to reactivation and depletion of the latent reservoir have been attempted clinically (summarized in [12,13]), and these efforts aim to reactivate latently infected cells so as to render them susceptible to viral cytopathic effects, an antiviral immune response, or other means of targeted cell killing [14,15]. However, complete depletion of the latent reservoir remains a long-term goal.

Although much attention is deservedly paid to defining how latency is maintained and how latent viruses can be reactivated, the mechanisms involved in the establishment of latency are incompletely understood. Given that the latent reservoir can be replenished during infection [16,17], a deeper knowledge of how latency is established would be invaluable. This review focuses on how HIV-1 latency is established at the cellular and molecular levels, and discusses potential approaches to limit the establishment of latent reservoirs.

### Establishment of HIV-1 latency at the cellular level

Although the pathways leading to latent virus reactivation can be studied *ex vivo*, it is not possible to study the establishment of latency in this manner, since by definition latency has already been established in any latently infected cells that can be isolated from an infected individual. Nonetheless, studies that investigate which subsets of resting cells harbor integrated virus in patients can be instructive, since knowledge of cellular physiology can shed light on how latent infection might have been established in a given cell type. Latently infected resting memory CD4 T-cells form the largest reservoir and represent the reservoir of greatest clinical importance due to their long lifespan [1]. Although it is likely that latency can occur in other cell types (reviewed in [1,18-20]), this review primarily focuses on the establishment of latency in CD4 T-cells.

#### Multiple CD4 T-cell subsets

Naïve CD4 T-cells are activated by interaction with dendritic cells (DC) that present an appropriate antigen. These activated T-cells then rapidly proliferate and differentiate into several subsets of effectors including Th1, Th2, Th17 and inducible regulatory T-cells [21]. While the majority of effector cells rapidly die, a small minority will survive and undergo a transition to a resting state as memory CD4 T-cells. Memory CD4 T-cells, which provide for an enhanced immune response upon future encounter with the same antigen, are likely derived from all effector subsets [22]. In addition, memory CD4 T-cells are themselves composed of several subsets that probably represent a gradient of separate maturational stages [23]. Central memory cells (T_CM_) migrate to secondary lymphoid organs where they can be activated by DCs to generate multiple waves of secondary effector cells. Effector memory cells (T_EM_) are likely derived from T_CM_, and are found in peripheral tissues, where they can act almost immediately as secondary effectors upon activation at sites of inflammation. Transitional memory cells (T_TM_) represent an intermediate cell type that possesses a phenotype intermediary between T_CM _and T_EM _[23-26]. Thus, the term “activated” CD4 T-cell can refer to either a primary effector cell that resulted from activation of a naïve cell, or to a secondary effector cell that resulted from activation of a memory cell. Similarly, the term “resting” CD4 T-cell can either refer to a naïve cell or to a memory cell. Resting cells can be distinguished from activated cells by their small size, low RNA content, non-cycling status, and lack of activation markers such as CD69, CD25 and HLA-DR [27].

#### Infection during deactivation vs. direct infection of resting cells

**Figure 1:**
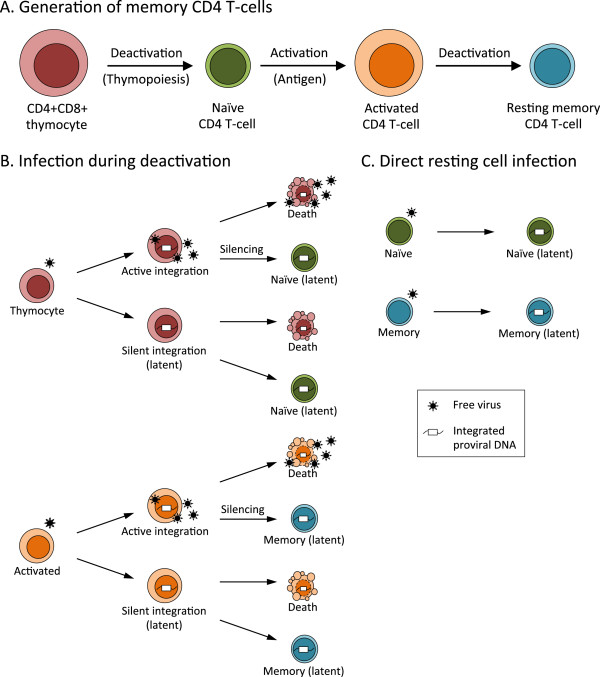
**Cellular pathways of the establishment of HIV-1 latency in CD4 T-cells.** (**A**) Generation of memory CD4 T-cells. Transcriptionally active CD4+CD8+ (double positive) thymocytes transition to a resting state upon completion of thymopoiesis to become resting naïve CD4 T-cells. Naïve cells are activated upon encounter with antigen-bearing dendritic cells and undergo rapid clonal expansion. A small fraction of activated CD4 T-cells survive and transition to a resting state, to become resting memory CD4 T-cells. (**B**) Infection during deactivation. Infection of an activated thymocyte can result in active integration or immediate silent integration. Latency can be established upon the transition to a naïve CD4 T-cell. Infection of an activated CD4 T-cell can result in active integration or immediate silent integration. Latency can be established upon the transition to a resting memory CD4 T-cell. Note that for immediate silent integration into an activated thymocyte or an activated CD4 T-cell, latency has already been established at the virological level. Due to the rapid deaths of activated cells, only cells which transition to a resting state represent clinically relevant latent infections. (**C**) Direct resting cell infection. Infection of a naïve CD4 T-cell, or of a resting memory CD4 T-cell, results in immediate silent integration, *i.e.,* latency. Note that the relative contributions of the pathways shown here are not known.

HIV-1 latency can arise in CD4 T-cells from infection of an activated effector cell that undergoes a reversion to a resting state during the process of memory cell generation (referred to herein as “infection during deactivation”), or from infection of a resting cell (direct resting cell infection), as illustrated in Figure 1. If latency is established during deactivation, then latent virus should be found mainly in memory cells. Conversely, direct infection of resting cells could result in latent virus being present in either naïve or memory cells. These pathways are not mutually exclusive. Latency can also be established during the deactivation process associated with thymopoiesis (discussed below), which would also result in latently infected naïve T-cells.

Infection of resting CD4 T-cells is inefficient due to many factors including low CCR5 expression [28], cytoskeletal barriers [29], limiting levels of deoxynucleoside triphosphates (dNTPs) [30,31] due to SAMHD1 [32,33], and inefficient nuclear import and integration [30,34]. *In vitro*, direct infection of naïve CD4 T-cells is less efficient than direct infection of memory CD4 T-cells [35,36]. This is because naïve cells have low to undetectable levels of CCR5 expression [28,37,38]; fusion is also less efficient in naïve cells [39], and cortical actin dynamics are lower compared to memory cells [40].

Several studies have examined the distribution of HIV-1 provirus in resting CD4 T-cells from peripheral blood and lymphoid tissues of patients. While some reports identified integrated DNA only in memory cells [41], most others have shown that memory cells constitute the major reservoir but that naïve cells harbour lower provirus levels [35,38,42-46]. In one recent study of patients on suppressive therapy, 98% of all provirus-containing CD4 T-cells were memory cells (of these, 52% were T_CM_, 34% were T_TM _and 14% were T_EM_), and only 2% were naïve cells [45]. In simian immunodeficiency virus (SIV)-infected rhesus macaques, most infected cells identified during early infection (*i.e.* the time of reservoir formation) were found to be resting CD4 T-cells [47]. Furthermore, cytokine/chemokine rich microenvironments in lymphoid tissues can aid infection of resting cells [48-51], and chemokine treatment of resting cells can lead to the establishment of latency *in vitro *[3,52,53]. It is, therefore, possible that the contribution of direct resting cell infection to the establishment of latency is greater than is commonly appreciated. Given that HIV-1 preferentially infects activated CD4 T-cells [30,34], coupled with the ongoing generation of memory cells, the consensus is that infection prior to or during deactivation is the major route of establishment of latency, although this remains an unresolved issue.

#### Routes of latency establishment: *in vivo* models

SIV-infected macaques receiving suppressive antiretroviral therapy are now excellent models to better understand the role of tissue reservoirs, sanctuary sites, viral dynamics in response to therapy, and *in vivo* testing of eradication strategies (reviewed in [54]). Humanized mouse models of HIV-1 latency are also useful and include severe combined immunodeficient humanized thymus/liver (SCID-hu Thy/Liv) mice [55], NOD/SCID-gamma chain null (NSG) bone marrow-liver-thymus (BLT) mice [56,57] and Rag2^−/−^γ_c_^−/−^ mice [58]. In SCID-hu (Thy/Liv) mice, latent infection is established during thymopoiesis, leading to generation of latently infected naïve T-cells. Thymopoiesis mirrors the generation of memory T-cells, since transcriptionally active immature CD4+CD8+ thymocytes enter a quiescent state upon maturation to naïve T-cells (Figure 1A). Therefore, the establishment of latency during thymopoiesis [55] is an example of latency arising from infection during deactivation. Latent virus was also identified in purified resting CD4 T-cells [57] and in naïve lymphocytes [56] of infected BLT mice, and in central memory CD4 T-cells of infected Rag2^−/−^γ_c_^−/−^ mice [58]. Collectively, these studies suggest that both infection during deactivation and direct infection of resting cells likely contribute to the establishment of latency *in vivo*.

#### Routes of latency establishment: *in vitro* models

Several primary cell latency models have been established (for detailed comparisons see [59-63]). Some of these models involve infection of activated CD4 T-cells that are allowed to return to a resting state through various culture conditions [64-69], with latency established in 1% to 75% of cells depending on the system. Several other models involve direct infection of either untreated or chemokine-treated resting CD4 T-cells [52,70-72] and result in up to a few percent of cells becoming latently infected, reflecting the preferential infection of activated cells. Taken together, these models demonstrate that both pathways can give rise to latency under appropriate conditions.

One report described the establishment of latency in multiple subsets of CD34+ hematopoietic progenitor cells (HPCs) derived from either bone marrow or umbilical cord blood [73]. In this model, purified HPCs are infected shortly after isolation and latency is established within a few days, in a manner analogous to direct infection of resting CD4 T-cells. Although the detection of HIV-1 DNA in HPCs from patients on suppressive highly active antiretroviral therapy (HAART) is controversial [74-77], it is clear that latency can be established in HPCs *in vitro *[73,75] (reviewed in [78]). While a latently infected HPC could theoretically give rise to other types of latently infected cells *in vivo*, including CD4 T-cells, it is unlikely that the virus would remain in a latent state during HPC differentiation [75].

Finally, a number of reports have described models of latency establishment at a population level in CD4 T-cell lines, including Jurkat [79-84], SupT1 [85,86] and Molt-4 [81] cells. The establishment of latency in proliferating cell lines implies that latency might be established in some fraction of infected, activated CD4 T-cells, even *in vivo* (included schematically in Figure 1B). However, the short lifespan of activated cells *in vivo *[87] implies that any such latent infections would be clinically irrelevant. Having examined how latency is established in terms of cellular physiology, we now turn our focus to the molecular level.

### Molecular mechanisms of the establishment of HIV-1 latency

The mechanisms associated with latency, particularly its maintenance and reactivation, have been extensively reviewed (for recent reviews see [63,88-91]). These mechanisms include transcriptional interference, insufficient levels of transcriptional activators, the presence of transcriptional repressors, epigenetics, nucleosome positioning, insufficient Tat activity, blocks to mRNA splicing or nuclear export, cellular microRNA (miRNA), and homeostatic proliferation of latently infected cells. While each of these is known to be involved in the maintenance of latency, here we discuss which of these mechanisms have been shown to promote viral entry into latency (summarized in Table 1). Homeostatic proliferation is an important mechanism of survival of resting CD4 T-cells that can be induced by homeostatic cytokines including IL-7 and IL-15 [92]. Since its role in maintaining latently infected cells occurs, by definition, after latency has been established, and in keeping with the focus of this review, homeostatic proliferation is not discussed here as a mechanism of establishment of latency.

**Table 1:** Mechanisms of latency establishment

**Mechanisms associated with latency**	**Evidence for a role in establishing latency in:**	
	**Cell line models**^**a**^	**Primary cell models**^**a**^
Transcriptional interference	Yes^b ^[68,102,104]	Yes [97]
Limiting transcription factors	Yes [81,84,106]*	Yes [81]
Limiting P-TEFb	?^c^	Yes [69,109]
Transcriptional repressors	?	?
Histone deacetylation	Yes [113]	Yes [69]
	No^d ^[81]	
Histone methylation	Yes [111,112,114,115]	Yes [69]
DNA methylation	No [81]	?
Nucleosome positioning	Yes [117]	?
Insufficient Tat activity	Yes [82,83,113,114,121]	Yes [69]
Insufficient mRNA nuclear export	?	Yes [3]
Insufficient mRNA splicing	?	Yes [4]
miRNA	?	?
Homeostatic proliferation	?	?

^a^ Only studies that explicitly examined the establishment of latency are included.

^b^ Yes: This mechanism has been shown to influence the establishment of latency.

^c^ ?: The effects of this mechanism on the establishment of latency have not been studied.

^d^ No: This mechanism has been shown to not influence the establishment of latency.

^*^ In [106] transcription factors may not be limiting, but altered the establishment of latency.

#### Transcriptional interference

HIV-1 preferentially integrates into the introns of actively expressed genes in CD4 T-cell lines [93,94], and both activated and resting primary CD4 T-cells that are infected *ex vivo *[95-97]. Initial studies in the Jurkat-based J-LAT system found that integration into both heterochromatin [79,98] and highly expressed genes [98] was associated with latency. Proviruses in resting CD4 T-cells from patients on HAART were also shown to be integrated into highly expressed genes, with no preference for orientation relative to the host gene [99]. A consequence of integration into regions of high transcriptional activity is transcriptional interference, a process whereby transcription that originates at one promoter can interfere with transcription at another (reviewed in [100,101]). One study found that convergently oriented integration resulted in transcriptional interference that silenced HIV-1 gene expression in a TNF-α-reversible manner [102], and similar findings were obtained in a Jurkat latency establishment model [68]. Another study found that transcriptional interference was responsible for latency in Jurkat and primary CD4 T-cells [103]. Transcriptional interference was also recently linked to the establishment of latency following viral integration into highly expressed genes in Jurkat cells, and the authors showed a role for chromatin reassembly factors in the maintenance of latency via transcriptional interference [104]. Finally, transcriptional interference contributed to the establishment of latency in a primary cell model, in which latent but not active proviruses had an orientation bias with respect to the host gene [97]. Although it is difficult to differentiate between roles for transcriptional interference in the establishment versus the maintenance of latency [81,104], most evidence suggests that both can occur depending on the host cell chromosomal context.

#### Limited availability of transcription factors

A hallmark of quiescent lymphocytes is the low availability of transcriptional activators, either due to cytoplasmic sequestration, or regulation of protein levels or activity. This includes the transcription factors NF-κB and NFAT, which recruit histone acetyltransferases [63] and aid transcription initiation, and are critical for viral transcription. Both NF-κB and NFAT are sequestered in the cytoplasm in the absence of activation signals, in part due to the protein Murr1 in the case of NF-κB [105]. In one study, the establishment of latency in Jurkat cells was found to result from low levels of active NF-κB at the time of infection, and only cell lines with low basal levels of NF-κB activity supported the establishment of latency. Furthermore, the induction of NF-κB nuclear translocation by pre-treatment of Jurkat cells with phorbol myristate acetate (PMA) or prostratin, or of primary cells with phytohemagglutinin (PHA), strongly inhibited the establishment of latency [81]. Another group found that Sp1 or κB site mutations (κB sites can be occupied by both NF-κB and NFAT) in the 5’ long terminal repeat (LTR) led to higher levels of latency [84]. In a model of latency establishment in CD34+ HPCs, nuclear levels of NF-κB were low at the time of infection but were increased upon stimulation and subsequent reactivation of latent virus [73].

It has recently been reported that the establishment of latency in a polyclonal population of Jurkat reporter cells was regulated by an AP-1 binding site in the 5’ LTR [106]. Deletion of this site severely limited the establishment of latency. Conversely, extension of this site from 4 to 7 nucleotides (as found in HIV-1 subtypes A and C) had no effect on initial latency levels but resulted in significantly greater levels of latency after several weeks of culture, likely due to lower rates of spontaneous reactivation of latent viruses carrying the 7 nucleotide sequence [106]. While this study does not necessarily provide evidence for a role of AP-1 in the establishment of latency, it suggests that variations in interactions involving transcription factors can have profound effects on the establishment of latency. Finally, it has been hypothesized that immunosuppressive cytokines including IL-10 and transforming growth factor beta (TGF-β) might indirectly aid the establishment of latency by reducing levels of T-cell activation [107], although this remains speculative.

#### Limited availability of elongation factors

The elongation factor P-TEFb is composed of Cyclin T1 and CDK9, and converts promoter-proximally paused RNA polymerase II complexes into efficient elongating complexes [90]. In many cell types P-TEFb is sequestered in the cytoplasm in a complex containing 7SK snRNA, HEXIM1, and other components [108], and a study using a primary cell latency model found that low P-TEFb levels contributed to latency establishment [69]. However, a recent study found that P-TEFb availability in both naïve and memory CD4 T-cells is regulated by tight control of Cyclin T1 levels (by proteasome-mediated proteolysis and microRNA regulation) and CDK9 T-loop phosphorylation (where only Thr-186-phosphorylated CDK9 is active), and not by the 7SK snRNA complex. The authors also showed that levels of Cyclin T1 and Thr-186-phosphorylated CDK9 decreased sharply during the transition of activated CD4 T-cells to central memory cells, during which time latency was established [109]. Thus, multiple mechanisms of transcriptional activator insufficiency can contribute to the establishment of latency.

#### Chromatin modifications

Epigenetic modifications dictate which proteins can interact with chromatin, and alter the physical structure of chromatin [110]. Proviral silencing after single-round infection of both Jurkat cells [111] and microglial cells [112] was shown to be mediated by the histone H3 lysine 9 (H3K9) methyltransferase Suv39H1 and its partner HP1γ. Entry into latency in Jurkat cells was associated with CBF-1-dependent histone deacetylase (HDAC)-1 recruitment to the 5’ LTR [113], and H3K9/27 trimethylation [114]. Furthermore, CBF-1-dependent H3 deacetylation, followed by Suv39H1- and HP1α-dependent H3K9/27 trimethylation, led to the establishment of latency in primary cells [69]. Interestingly, CBF-1 is expressed in resting CD4 T-cells but is strongly downregulated upon T-cell activation [113]. Most recently, this group has demonstrated a role for the H3K27 methyltransferase EZH2, a component of the polycomb repressive complex 2, in establishing latency in Jurkat cells [115]. However, a different study found no evidence for histone deacetylation in the establishment of latency, since pre-treatment of Jurkat cells with the HDAC inhibitor valproic acid did not reduce the number of latently infected cells that were established [81].

DNA methylation at CpG islands is a repressive epigenetic modification that can inhibit transcription factor binding and can recruit HDAC-2. The available evidence suggests that DNA methylation is a later silencing event that is more important for the maintenance of HIV-1 latency than for its establishment [89,116]. Additionally, one study showed that pre-treatment of Jurkat cells with the DNA methylation inhibitor 5-azacytidine did not inhibit the establishment of latency [81]. Finally, the SWI/SNF chromatin remodeling complex BAF, but not PBAF, was recently shown to facilitate the establishment of latency through repressive nucleosome positioning on the 5’ LTR. BAF knockdown resulted in fewer latent infections in both Jurkat and SupT1 T-cell lines, without affecting levels of productively infected cells [117]. The evidence therefore supports a major role for epigenetic histone modifications and chromatin remodeling leading to provirus silencing and the establishment of latent infection.

#### Insufficient Tat activity

Since Tat is required for high-level viral transcription, due to recruitment of a super elongation complex to the 5’ LTR [118,119], it is perhaps unsurprising that insufficient Tat activity can lead to the establishment of latency. In one study, resting CD4 T-cells from treated patients were enriched for attenuated Tat variants [120]. Mutations that attenuated Tat activity led to higher levels of latency establishment in both Jurkat [82,113,114] and primary cell [69] models. Treatment of Jurkat cells with Tat at the time of infection led to a subsequent decrease in the frequency of latently infected cells [82]. Further, expression of Tat in *trans* prevented the silencing of actively infected cells [114] and strongly inhibited the establishment of latency in Jurkat cells [82]. Finally, random fluctuations in Tat concentrations at the single cell level were shown to influence the entry of HIV-1 into latency, as shown in mathematical models and experimentally [83,121]. Based on these findings, proteins that modulate Tat activity might be expected to impact the establishment of latency, as has been suggested for Tat acetylation via SirT1 [121].

#### Post-transcriptional mechanisms

Multiply spliced mRNA was found in the nucleus, but not in the cytoplasm, of resting CD4 T-cells from HAART-treated patients. This block was shown to be due to low levels of polypyrimidine tract binding protein (PTB), the overexpression of which rescued multiply spliced mRNA nuclear export and virus production [2]. However, it was unclear whether limiting PTB levels contributed to the initial establishment of latency. In a primary cell model in which resting cells are directly infected after chemokine treatment [52], it was shown that multiply spliced mRNA accumulated in the nucleus but not the cytoplasm, in the absence of other transcripts or viral proteins [3]. In another resting cell model of latency establishment, [70] a block to mRNA splicing was recently identified, whereby latently infected cells produced Gag protein (at levels 1000-fold lower than in activated cells) but only barely detectable levels of Env. This result was reflected at the mRNA level, since unspliced transcripts were ~100-fold more abundant than singly spliced transcripts and ~10,000-fold more abundant than multiply spliced transcripts [4]. Together, these primary cell models highlight two post-transcriptional blocks that contribute to the establishment of latency. In addition, miRNA regulation of viral protein expression has been associated with latency, and several of the miRNAs that have been implicated in this process are expressed in resting cells but are downregulated upon T-cell activation. Although miRNAs can contribute to the maintenance of latency, as shown both *in vitro* and *ex vivo*[122,123]*,* the potential role of miRNAs in the establishment of latency remains unknown [124].

#### Silencing of active infection vs. immediate silent integration

It is unclear whether latency is established by the silencing of active infection or by the immediate silent integration of viral DNA (Figure 1B-C). Several *in vitro* studies have examined these alternatives, and additional information can be gathered from close analysis of cell culture models of latency establishment. First, it should be noted that evidence in favor of one route of latency establishment does not necessarily exclude the other. Some Jurkat [114] and primary cell [67,69] models involve cell sorting for active infections that are then cultured and allowed to become latent, demonstrating that the silencing of active infections can lead to the establishment of latency. In these reports some viral proteins were mutated to prevent their expression, resulting in reduced cytotoxicity, which might have allowed cells to survive long enough in order for silencing to occur. One study provided evidence for silencing of active infections in both CEM and primary cells, without the use of cell sorting and with replication-competent virus [125].

Several other groups have provided evidence for immediate silent integration. For example, J-LAT cells were derived by sorting GFP-negative cells shortly after infection with a reporter construct [79]. Additional studies in CD4 T-cell lines have provided evidence for silent integration leading to latency, sometimes by showing reactivation of latent virus as early as one day post-infection [81,82,86,106,126]. Data from a primary cell model in which cells are infected during the transition to a resting state suggest that latency occurred largely by silent integration [66]. Finally, all published latency models that depend on direct infection of resting cells have shown immediate silent integration [3,70-72]. Thus, silencing of active infection, and immediate silent integration, both contribute to the establishment of latency *in vitro*, and direct infection of resting cells consistently results in immediate silent integration. If, however, the majority of latent infections *in vivo* arise from infection prior to or during cellular deactivation, the pathway of latency establishment is likely to depend on how far along the deactivation process a given cell is at the time of infection.

### Prospects for inhibition of the establishment of latency

Depletion of the latent reservoir is a major goal of the field, and this might be complemented by strategies aimed at limiting the establishment of latent infections. Whether the establishment of latent reservoirs can be inhibited in patients is an important issue in the quest for a functional cure [127]. This has been examined *in vitro*, through studies in which treatment of Jurkat cells with exogenous Tat protein led to a reduction in the establishment of latency [82]. A novel approach has recently been proposed which would involve interference with chemokine-induced establishment of latency. In this scenario, treatment with chemokine receptor antagonists or engineered ‘dominant negative’ chemokines would inhibit the establishment of latent infections that result from direct infection of resting cells [128]. Several clinical studies have observed that very early initiation of HAART can lead to the establishment of smaller latent reservoirs than are observed if treatment is started later [7,45,129-132]. It remains to be determined whether early treatment with compounds that counteract pathways of the establishment of latency merits clinical consideration.

## Conclusions

The establishment of HIV-1 latency is a complex process, which likely results from the convergence of multiple mechanisms (Table 1). The relative importance of these mechanisms is probably dependent on the physiological state of the cell undergoing infection (Figure 1). Are findings in cell line models of establishment of latency reproducible in primary cell models? Although they often are, it also appears that not all mechanisms involved in the establishment of latency play a role in cell lines. For example, it has been proposed that epigenetic silencing might have a greater role in cell lines than in primary cells [71], since several other mechanisms of establishment of latency, including limited availability of transcription factors, P-TEFb, and the nuclear export factor PTB are mainly associated with quiescent cells and might be less important in actively dividing cells. Are different mechanisms of silencing required depending on the pathway of latency establishment, *i.e.* infection during deactivation vs. direct resting cell infection, or latency resulting from silencing of active infection vs. immediate silent integration? Although this is an open question, the evidence suggests that these different routes of establishment of latency can all occur under different circumstances.

It is not yet known whether the establishment of latency might differ between memory CD4 T-cell subsets, for example in T_CM _compared to T_TM_. Additionally, little is known about how latency can be established in other cell types, which might exhibit important differences compared to CD4 T-cells. It is also unclear how well the different models of latency establishment recapitulate this process in patients. Which primary cell model(s) might reflect the *in vivo* establishment of latency most accurately? The answers to this and related questions await a better understanding of the mechanisms and routes of latency establishment under *in vivo* conditions. Finally, the feasibility of pharmacological interventions that would limit the establishment of latent reservoirs, and any potential clinical benefits this might entail, remain important unanswered questions.

## Competing interests

The authors declare that they have no competing interests.

## Authors’ contributions

DAD wrote the manuscript. MAW modified parts of the manuscript in his role as Head of the Laboratory. Both authors read and approved the final manuscript.
